# Biplanar High-Speed Fluoroscopy of Pony Superficial Digital Flexor Tendon (SDFT)—An In Vivo Pilot Study

**DOI:** 10.3390/vetsci8060092

**Published:** 2021-05-27

**Authors:** Franziska C. Wagner, Kerstin Gerlach, Sandra M. Geiger, Claudia Gittel, Peter Böttcher, Christoph K. W. Mülling

**Affiliations:** 1 Institute of Veterinary Anatomy, Histology and Embryology, Faculty of Veterinary Medicine, Leipzig University, An den Tierkliniken 43, 04103 Leipzig, Germany; sandra.m.geiger@web.de (S.M.G.); c.muelling@vetmed.uni-leipzig.de (C.K.W.M.); 2 Department for Horses, Faculty of Veterinary Medicine, Leipzig University, An den Tierkliniken 43, 04103 Leipzig, Germany; gerlach@vetmed.uni-leipzig.de (K.G.); csg39@cam.ac.uk (C.G.); 3 Small Animal Clinic, Department of Veterinary Medicine, Freie Universität Berlin, Oertzenweg 19b, 14163 Berlin, Germany; peter.boettcher@fu-berlin.de

**Keywords:** horse, equine, gait, collagenase, XROMM, strain, tendinopathy

## Abstract

The superficial digital flexor tendon (SDFT) is the most frequently injured structure of the musculoskeletal system in sport horses and a common cause for early retirement. This project’s aim was to visualize and measure the strain of the sound, injured, and healing SDFTs in a pony during walk and trot. For this purpose, biplanar high-speed fluoroscopic kinematography (FluoKin), as a high precision X-ray movement analysis tool, was used for the first time in vivo with equine tendons. The strain in the metacarpal region of the sound SDFT was 2.86% during walk and 6.78% during trot. When injured, the strain increased to 3.38% during walk and decreased to 5.96% during trot. The baseline strain in the mid-metacarpal region was 3.13% during walk and 6.06% during trot and, when injured, decreased to 2.98% and increased to 7.61%, respectively. Following tendon injury, the mid-metacarpal region contributed less to the overall strain during walk but showed increased contribution during trot. Using this marker-based FluoKin technique, direct, high-precision, and long-term strain measurements in the same individual are possible. We conclude that FluoKin is a powerful tool for gaining deeper insight into equine tendon biomechanics.

## 1. Introduction

Injuries of the superficial digital flexor tendon (SDFT), especially in the metacarpal region, are a common musculoskeletal disorder in sport horses [1,2,3,4]. Tendonitis of the SDFT also represents the most common cause of lameness and early retirement in ponies used for sport, such as polo ponies [5]. The convalescence period after tendinopathy lasts approximately nine to eighteen months [6]. Within a follow-up period of two years, 40% of those horses suffer re-injury [7]. Human Achilles tendonitis is a comparable pathophysiological condition to the disorders of equine SDFT, which is considered a good animal model also because of similar physiology [8,9]. Due to being “spring-loaded”, the SDFT operates near its physiological limit under maximal load [10,11,12,13]. In a galloping horse, strains of up to 16.6% have been measured in the SDFT [14].

Different techniques are used to measure the strain of tendons. The invasive nature of Hall effect transducers, as well as mercury strain gauges, is detrimental as the devices are relatively bulky and the exact calibration of the measurement tool needs additional ex vivo tests and therefore euthanasia of the animal [14,15]. Several noninvasive techniques for evaluating tendon biomechanics have been developed, including elastography, measurement of the axial speed of sound, and acoustoelastography [16,17,18,19]. These methods entail mathematical calculation of models and are therefore indirect approaches to measure tendon strain. Strain can also be calculated from joint angulation and ground reaction forces [20] or be estimated based on the elastic modulus of a tendon segment from its mean echogenicity [21].

Using FluoKin, a three-dimensional X-ray system retrofitted with high-speed cameras (XROMM) for direct motion analysis is well described in Brainerd et al. [22]. Transferring this radiographic method from bone to soft tissue, the implantation of radio-opaque markers into soft tissue has been described in the cruciate ligament [23], the human Achilles tendon [24], the rotator cuff [25], and muscle tissue [26]. Wagner et al. have demonstrated the ability of this technique to measure the strain of the SDFT ex vivo, by calculating intermarker distances of implanted tantalum beads [27]. To date, FluoKin has not been used for in vivo assessment of the SDFT. The X-ray system used in the present study can reach a precision of 0.043 mm under optimal conditions and 0.055 mm in the moving SDFT [27].

The aim of the present long-term study was to assess the SDFT in vivo in one pony, prior to and following tendon injury. For this purpose, FluoKin and ultrasound were used to evaluate strain and tendon structure. We expected a difference in strain between walk and trot as well as under sound, injured, and healing conditions. We hypothesized that in the healing SDFT, FluoKin would be able to detect remaining biomechanical weakness despite clinical and ultrasound examination suggesting normal and fully functional tendon tissue.

## 2. Materials and Methods

### 2.1. Study Design and Animal

**Figure 1:**
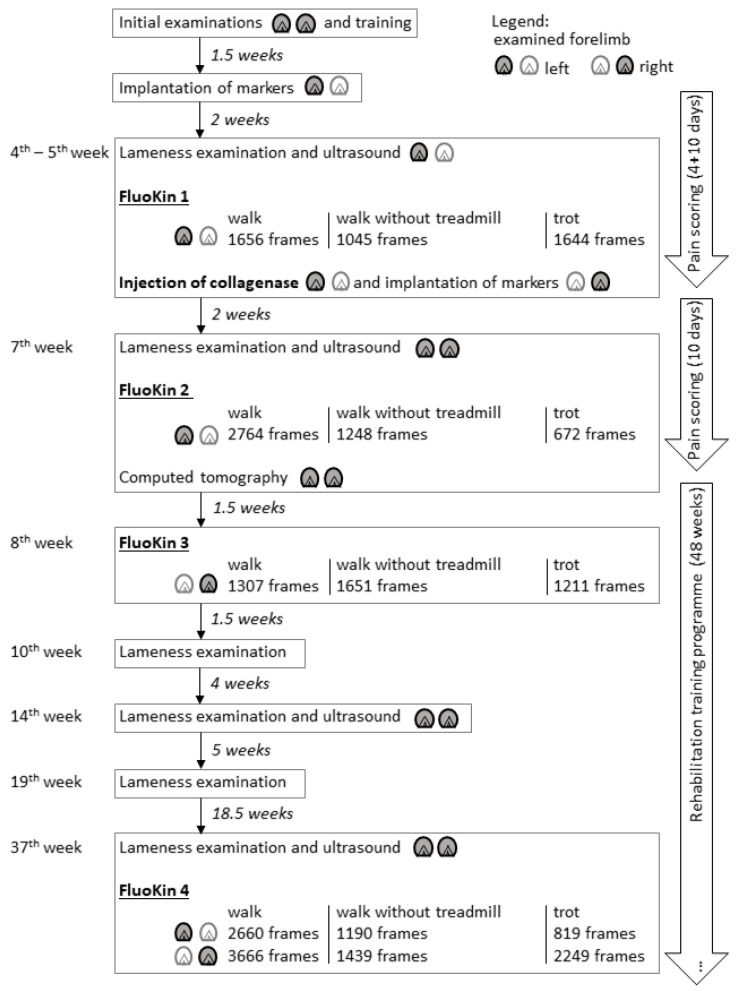
Time schedule of the experimental set-up. Additionally, short clinical examinations were carried out every day within the clinic’s routine.

This prospective pilot study was approved by the federal ethics committee (Landesdirektion, Leipzig, Germany, No. TVV 20/16). A seven-year-old Shetland pony gelding was used as a pilot animal to test the transferability to living animals, of the methods formerly tested with ex vivo tensile tests of forelimb SDFT [27]. A Shetland pony was used as the length of its metacarpus can be video-radiographed completely in one FluoKin measurement. The animal was kept together with another Shetland pony and had access to a paddock. Daily clinical examinations were carried out within the clinic’s routine. The pony was trained before the gait recordings so that it would walk in the gait lab without hesitation. Implanted radio-opaque markers were used to monitor the strain of the SDFT during walk and trot with FluoKin. FluoKin examinations were carried out before and after induction of a tendon lesion (Figure 1).

### 2.2. Implantation of Markers

**Figure 2:**
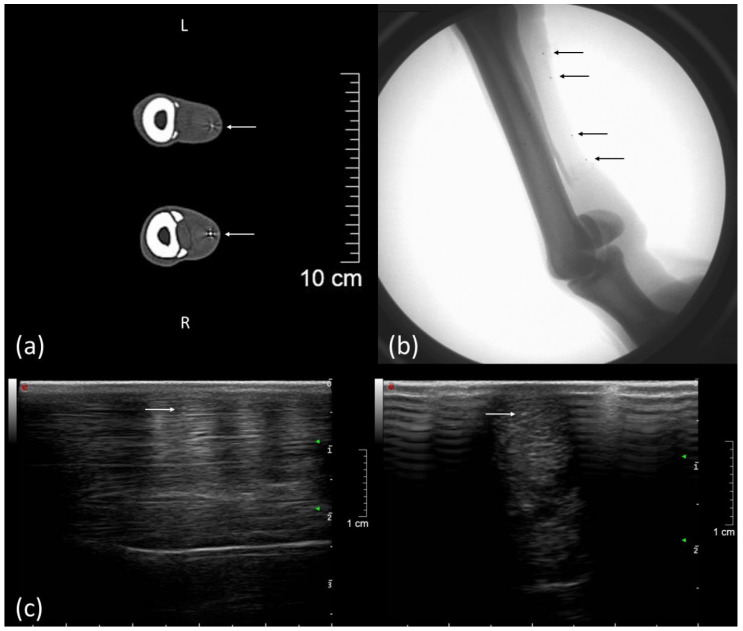
Computed tomography (CT), FluoKin, and ultrasonographic images of the left forelimb of a pony with implanted tantalum beads. (**a**) Two tendon markers with metal streak artifact in the CT image, left is dorsal; (**b**) FluoKin image of the distal forelimb with four tendon markers in the SDFT and five bone markers in the third metacarpal bone; (**c**) longitudinal and transversal ultrasound image with acoustic shadowing and reverberation artifact due to the implanted beads, left is distal. Arrows mark the implanted beads.

**Figure 3:**
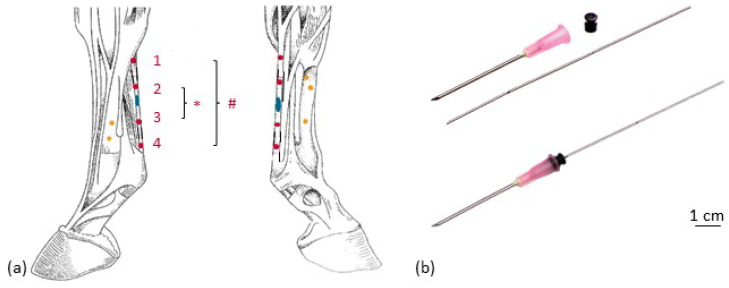
Implantation of the tantalum beads. (**a**) Left forelimb, lateral (left) and medial (right) view; yellow: bone markers, red: tendon markers with intermarker distances M1 to M4 (#) and M2 to M3 (*); blue: collagenase. (**b**) Implantation instrument for tendon markers (separated and mounted).

To visualize tendon kinematics, four tantalum beads (0.8 mm diameter, X-medics, Scandinavia, Frederiksberg, Denmark) were implanted in the tendon tissue of the metacarpal region of the SDFT. Additionally, five tantalum beads (1.0 mm diameter, X-medics, Scandinavia, Frederiksberg, Denmark) were implanted in the third metacarpal bone (Figure 2) in order to visualize SDFT kinematics and bone movement simultaneously (Vedio S1). In order to be able to assess the influence of the implanted markers on the gait pattern, or the possible lameness induced, the markers were initially implanted only in one forelimb (left). The procedure was performed under general anesthesia after aseptic preparation of the implantation sites according to the following protocols: For the tendon implantation, two skin incisions of 2 cm each were made palmarly in the cranial and distal aspect of the metacarpal region. With the aid of an 18 G cannula (Becton Dickinson, Franklin Lakes, NJ, USA) and an inserted custom-made mandrin, two beads per insertion were placed within the tendon. The mandrin was secured in the cannula with the piece of elastic from an insulin syringe. Two milled marks at the mandarin prevented (1) pulling it out too far before injecting the bead and (2) pushing it too deep into the tendon tissue while implanting the bead (Figure 3). To implant the bone markers, the skin was incised 5 mm (see Figure 2), and a bone marrow mandrin (Somatex Medical Technologies GmbH, Berlin, Germany) was placed. A 1.6 mm hole was drilled in the cortical bone with an orthopedic drilling machine (Colibri II, DePuy Synthes, Umkirch, Germany). Each bead was fixed by slightly hammering on a bone marrow trephine needle. The skin was sutured and disinfected with antiseptic iodine spray, and the operation area was bandaged until the skin sutures were removed 11 days after operation. The operation area was checked according to a pain scoring system [28,29].

### 2.3. Injection of Collagenase

In a second setting, two weeks after the implantation of markers into the left forelimb, tantalum markers were implanted in a similar manner in the right forelimb. At the same time, collagenase was injected in the SDFT of the left forelimb to create an idiopathic tendon lesion. Collagenase I (Life Technologies GmbH/Thermo Fisher Scientific, catalog number 17100017, Darmstadt, Germany) at a concentration of 4.8 mg/mL was injected in the metacarpal region of the left SDFT in a total volume of 0.1 mL (mixed with Hank’s Balanced Salt Solution) (Figure 1 and Figure 2). After that, a rehabilitation training program of 48 weeks was performed according to Smith and McIllwraith [29].

### 2.4. Examinations and Treatment

Initial examinations of the pony included a clinical examination, a large blood count, a lameness examination, an X-ray examination (Gierth X-ray International GmbH, HF 1000/MERS 1005, Riesa, Germany) of the distal forelimbs, and an ultrasound examination of the palmar tendons and of the common extensor tendon (MyLabTwice, Esaote Biomedica Deutschland GmbH, Köln, Germany). The ultrasound examination was performed with a linear array ultrasonic transducer at 3 cm penetration depth. No pathologies were found. Pain scoring according to Bussières et al. [28] and Dalla Costa et al. [30] was performed daily, starting 4 days before surgery (baseline) and 3 times a day for a period of 10 days after operation. After implanting markers in both forelimbs, an ultrasound examination and a computed tomography (CT) of the metacarpal region (Philips Mx 8000 IDT, Philips Healthcare, DA Best, Netherlands, slice thickness of 0.8 mm) confirmed the marker positions in the bone and tendon (Figure 1 and Figure 3). All lameness examinations and ultrasound of the SDFT, as shown in Figure 1, were performed by equine veterinarians experienced in orthopedics. The ultrasound examinations were carried out by the same person (PD Dr. Gerlach).

The pony was given a single dose of benzyl penicillin Na IV (2.2 mg/kg body weight) intra-operation. Flunixin meglumine (Flunidol, CP-Pharma Handelsgesellschaft mbH, Burgdorf, Germany) was administered P.O. as pain medication: 1.1 mg/kg body weight on the operation day, 0.55 mg/kg body weight b.i.d. on days 1 to 4 *post operationem* and 0.55 mg/kg body weight on days 5 and 6 *post operationem*.

### 2.5. Biplanar High-Speed Fluoroscopy

The biplanar X-ray motion analysis was conducted at the FluoKin gait lab of Leipzig University, which has been described in detail by Weiss et al. [31] and Geiger et al. [32]. Continuously, X-ray videos were taken from two directions, enabling the reconstruction of the movement of the video-radiographed object in all six degrees of freedom. After two weeks of stable rest, after each implantation, FluoKin videos were taken while the pony walked and trotted on the treadmill and at walk without treadmill, by walking the pony through the image field. At least two steps in walk (with and without treadmill) and in trot were recorded. FluoKin examinations were performed on healthy forelimbs (left in “FluoKin 1” and right in “FluoKin 3”), with collagenase-induced lesion in the left SDFT (“FluoKin 2”), and 33 weeks after injection of collagenase (both forelimbs in “FluoKin 4”) (Figure 1). The set-up of the gait lab with the treadmill between the X-ray sources and the image intensifiers required an interbeam angle of approximately 60° and a source–image distance of 140 cm. The metacarpal region was imaged at 46–50 kV and 80 mA, with 0.5 ms shutter speed, without magnification. Videos were recorded for 6 s with 500 fps in a resolution of 1024 × 1024 pixels. Undistortion, calibration, and marker tracking were performed with XMALab (version 1.3.8, www.bitbucket.org/xromm/xmalab, download 30 September 2016) according to Knoerlein et al. [33]. The tracked marker positions were manually refined after automatic marker tracking and a low-pass, 5th-order, Butterworth filter at 35 Hz. The mean reprojection error of the tendon markers was 0.3265 pixels (1st quartile: 0.2120 pixels; 3rd quartile: 0.4311 pixels; maximum: 0.9550 pixels; minimum: 0.0885 pixels). Tendon strain was calculated from the intermarker distance between the pairs M1 to M4 and M2 to M3 of the implanted tantalum beads (see Figure 3). Statistical examination was carried out with matched-pairs t-tests over each condition. Results were considered to be significant when *p* < 0.05.

## 3. Results

### 3.1. Clinical Examination Including Ultrasound and Computed Tomography

Tiny tantalum beads (0.8 mm), as tendon markers in the SDFT, can be palpated at the unloaded limb without provoking a defensive movement. No pain or lameness was detected after marker implantation in the SDFTs or metacarpal bones. No inflammation of the tendon tissue, caused by the marker implantation, could be detected via ultrasound. The implanted beads could clearly be identified with ultrasound imaging and CT and in the FluoKin videos (Figure 2). The tantalum beads produced reverberation artifacts and an acoustic enhancement in ultrasound images and metal artifacts in computed tomography images.

**Figure 4:**
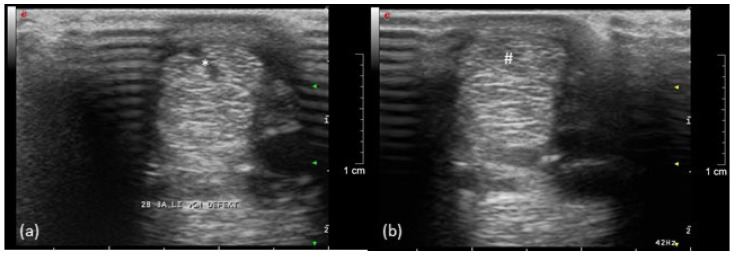
Ultrasonographic image of the left forelimb of a pony, left is lateral: (**a**) acute (*) and (**b**) healing (#) tendon lesion of the SDFT (9 weeks after injection of collagenase).

A mild swelling occurred for several weeks in the bone marker regions with no other signs of inflammation or pain during palpation. During the lameness examination, in the 14th week, the implantation sites were no longer circumferentially enlarged. After walking and trotting on the treadmill during the first FluoKin measurement (“FluoKin 1”), a diffuse swelling of the lateral aspect of the metacarpal region appeared for three days. Two weeks after injection of collagenase, the pony showed a lameness score of 2 out of 5 in the left forelimb during walk and trot but maintained an even weight distribution while standing. The corresponding ultrasound examination of the left forelimb SDFT showed an acute, hypoechogenic tendon lesion in the center of the mid-metacarpal region with a slightly enlarged diameter (Figure 4). In the right forelimb, no signs of tendonitis were seen in the ultrasound in the 7th week. Five weeks after injection of collagenase, no lameness but a slightly more careful use of the left forelimb was observed during walk. There was a lameness score of 1 out of 5 during trot. Nine weeks after injection of collagenase, no gait abnormalities during walk and a barely noticeable lameness score of 1 out of 5 during trot were detected. Within the corresponding ultrasound examination in week 14, hyperechogenic lines in the tension direction were demonstrated in the remaining hypoechogenic lesion (Figure 4). Fifteen and thirty-three weeks after injecting collagenase, no lameness could be observed. The final ultrasound examination in the 37th week showed normal tendon tissue. The beads did not slip out of the tendon over the course of the study. The proper location of all beads in the tendon tissue was verified both with CT in week 7 and repetitive ultrasound examinations (4th week, 7th week, 14th week, and 37th week).

### 3.2. Biplanar High-Speed Fluoroscopy

**Figure 5:**
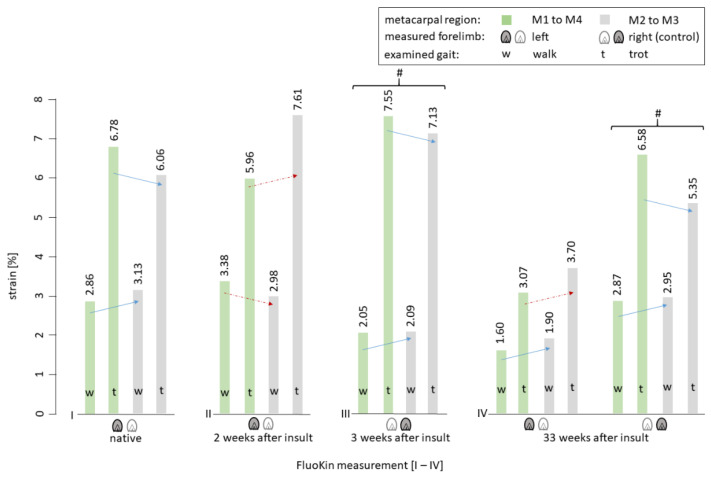
Bar graph of the measured strain of the SDFT of both forelimbs during walk and trot between the implanted tendon markers. Blue arrows: strain behavior in the sound SDFT (right forelimb and “native”). Red dotted arrows: alternated strain behavior compared to “native” and measurements within the right (sound) forelimb. # indicates nonsignificant differences between corresponding measurements in the right, not-insulted forelimb.

**Figure 6:**
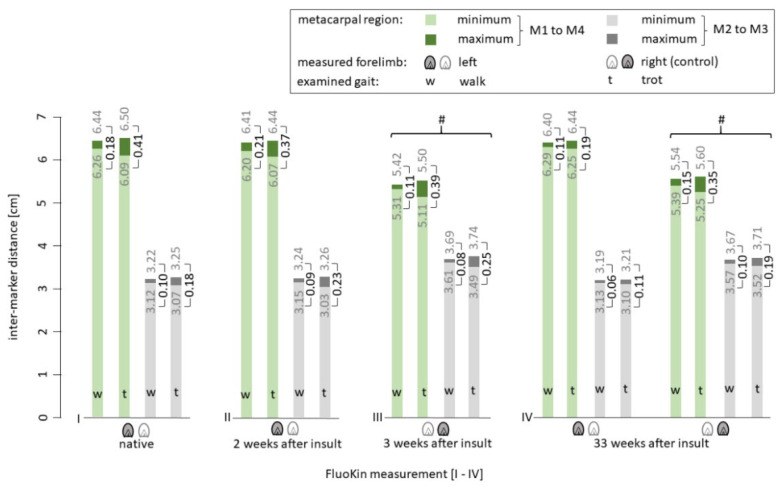
Bar graph of the length variation of the tendon markers of both forelimbs during walk and trot. # indicates nonsignificant differences between corresponding amplitudes in the right, not-insulted forelimb.

During the in vivo FluoKin measurements, the pony walked and trotted on a treadmill with its forelimbs positioned in the biplanar X-ray image field. Length variations between the four implanted tantalum beads in the SDFT (see Figure 1) were measured and strain was calculated. Differences in strain between walk and trot were significant in all measurements (Figure 5) (*p* < 0.01). This is caused by a more pronounced recoil of the tendon and a greater maximum stretch during trot than during walk: during trot, the minimal intermarker distance was significantly smaller (M1 to M4: −0.1365 ± 0.0573 cm with *p* < 0.01; M2 to M3: −0.0753 ± 0.0427 cm with *p* < 0.02) while the maximum intermarker distance was significantly larger (M1 to M4: +0.0525 ± 0.0213 cm with *p* < 0.01; M2 to M3: +0.0320 ± 0.0132 cm with *p* < 0.01) than during walk (see Figure 6). There was no significant difference between measurements during walk with and without the treadmill (M1 to M4: 0.0145 cm ± 0.0503 and 0.2896% ± 0.8900; M2 to M3: 0.0139 cm ± 0.0226 and 0.3993% ± 0.6945).

In the second FluoKin measurement, two weeks after induction of tendinopathy with collagenase injection, the maximum intermarker distance M1 to M4 (representing the whole metacarpal region) was shorter than the values in the sound condition (−0.0243 cm during walk, −0.0640 cm during trot). In contrast, the intermarker distance of the mid-metacarpal region M2 to M3 reached higher maxima when injured (+0.0244 cm during walk, +0.0051 cm during trot). The fourth FluoKin measurement was 33 weeks after the insult. Compared to the sound (FluoKin 1) and injured (FluoKin 2) measurements, the intermarker distances (cm) and tendon strain (%) were lower in the metacarpal (M1 to M4) and mid-metacarpal (M2 to M3) regions, during both walk and trot. The differences during walk were as follows: M1 to M4: −0.08 cm, −0.11 cm and −1.26%, −1.78%; M2 to M3: −0.04 cm, −0.03 cm and −1.23%, −1.08%. The differences during trot were as follows: M1 to M4: −0.22 cm, −0.17 cm and −3.71%, −2.89%; M2 to M3: −0.07 cm, −0.12 cm and −2.37%, −3.91%. Additionally, in the fourth FluoKin measurement, compared to the first, maximum intermarker distances were smaller (M1 to M4: −0.0422 cm during walk, −0.0595 cm during trot; M2 to M3: −0.0263 cm during walk, −0.0406 cm during trot), while minimum intermarker distances were higher (M1 to M4: +0.0359 cm during walk, +0.1614 cm during trot; M2 to M3: +0.0120 cm during walk, +0.0310 cm during trot).

In the two measurements of the right forelimb (“FluoKin 3” and “FluoKin 4”), which was used as a control for the sound condition, no significant differences in intermarker distances and strain could be detected (Figure 5 and Figure 6).

## 4. Discussion

The present pilot study demonstrates the successful use of FluoKin to detect strain of the SDFT in vivo in a Shetland pony and to track the development of a collagenase-induced tendon lesion. To measure the length variation of the SDFT during movement with an X-ray technique, 0.8 mm diameter tantalum markers had been implanted into the SDFT of both forelimbs and the third metacarpal bones.

### 4.1. Clinical Examination

The pony was monitored for ten days after each surgery, i.e., for implantation of the markers and also after injection of collagenase. For pain scoring in animals, it is recommended to use validated scoring systems [34]. We used a composite pain score [28] and a facial expression pain score [30] to have a multidimensional pain assessment. No pain was detected by any of the three unvarying observers at any of the three observation times, on any day. While Bussières et al. [28] developed and validated their pain scale in horses and not ponies, Van Loon and Van Dierendonck [35] found no differences in the ability to recognize pain between the two. Even though many tendon lesions are not painful [36], a nonsteroidal anti-inflammatory drug (which also reduces pain) was given in the present study over six days after surgery to regulate the initial inflammatory response, which is beneficial for tissue repair [37]. Further, controlled motion is crucial for flexor tendon healing [38], which is why the pony was trained according to Smith and McIllwraith [29].

There was a slight tissue swelling above the implantation site of three of the five bone markers, which was probably due to a mild reaction to the surgery. A diffuse nonpainful swelling at the lateral metacarpal site occurred for three days after the first FluoKin measurement (“FluoKin 1”), probably due to the movement required for the measurement. This could be due to a delay in lymphatic drainage due to the previous surgery. Over the whole study term, all beads remained inert in the tissue as expected [39,40], with no migration out of the tendon tissue occurring. It is assumed that migration could potentially occur within the first six weeks after implantation [41].

### 4.2. Ultrasound Examination

The location of the implanted tantalum beads with ultrasound required practice, whereas the markers in the metacarpal bones and SDFTs could be easily located with CT and FluoKin. Tendon healing follows the general principles of wound healing: after an inflammation phase of a few days (4 to 10), there is a proliferation phase of a few weeks (4 to 45 days) and a remodeling phase (45 to 120 days) [42,43,44]. Echogenicity is decreased in SDFT lesions [21] due to fiber destruction and inflammation, as also shown in our study. Echogenicity progressively increases during the healing process according to fiber realignment [45]. Realignment of collagen fibers along the force direction can be seen after 8 to 12 weeks. They are transformed into a scar-like tissue, and after 17 weeks the ultrasonographic appearance of the tendon bundles is similar to that of normal tendons [46]. In our study, hyperechogenic lines, which are probably related to scar tissue, were seen nine weeks after injection of collagenase. The healing process in ponies is faster than in horses due to a higher activity of leucocytes and a more intense initial inflammatory response [47,48]. This is also described in severe limb injuries with tendon ruptures and periosteal exposure [48]. In the present study, ultrasound examinations were always conducted by the same experienced veterinarian to exclude variation in the evaluation [49]. However, ultrasonography in general can underestimate the size of tendon damage, especially in long-term injuries [50]. Thirty-three weeks after the injury, no evidence of a lesion could be detected by ultrasonography, even in off-angled views. In the right forelimb, no lameness or signs of tendonitis were found, whereas tendon pathology in the contralateral limb is common [51].

### 4.3. Biplanar High-Speed Fluoroscopy

Biplanar high-speed fluoroscopy (FluoKin) is a 3D X-ray analysis system. The three-dimensionality of the measurements is achieved by using two X-ray units retrofitted with high-speed cameras. For measuring strain distribution of the SDFT with FluoKin, we implanted four tantalum beads along the tendon (Figure 1). Crevier et al. [52] demonstrated that strain is not equally distributed along the tendon. The bead position remained stable in the tendon tissue during the 35 weeks of experiment. Soft tissue markers are encapsulated within two weeks and, thereafter, are expected to remain in a stable position [53]. We used the same implantation protocol for the tendon markers as reported previously by Wagner et al. [27] and proposed by Smith et al. for the bovine anterior cruciate ligament [54]. Injecting the beads across the fiber direction avoids migration along the injection canal during tensile strain, as validated in an ex vivo experiment [54]. During the initial healing phase, following the implantation, the pony underwent stable rest, with the first FluoKin measurements performed two weeks after surgery. Following the subsequent proliferation phase, fibrosis around the tantalum beads would be expected to hold them stable in their position. Several FluoKin studies have shown that markers do not migrate out of the cortex of the bone [31,32,55,56]. This might due to slightly hammering the markers into the drilled hole in the cortex of the bone, as performed in the former studies as well as in the present study to prevent marker migration. Due to the bio-inertness of tantalum [40], the beads can remain in the tissue, without reaction, for a lifetime [57].

There are several advantages to using FluoKin in tendon strain studies. The measurements are of high precision and cover a larger region at the same time of data acquisition. Further, the movement of both the tendon and bone can be visualized simultaneously. To confirm that small variations in tendon strain can be detected, we previously validated the FluoKin lab by measuring a known distance between pairs of tantalum beads embedded in an aluminum sheet and implanted in a frozen pony forelimb [27]. In that study, a precision of 0.0553 mm and 0.14° in SDFT and 0.0985 mm and 0.16° in the third metacarpal bone was found in dynamic measurements. Markers are distributed as widely as possible in the third metacarpal bone as proposed by Brainerd et al. [22]. On the contrary, linearity is desired while implanting the beads into the SDFT. To increase the interindividual comparability, it would even be desirable to be able to implant the tendon markers at a safely defined location. Not perfectly aligned markers could be compensated using virtual markers in the 3D animation, which would allow the measurement of length variation along the tendon line of action. With manual implantation, the achieved accuracy is most likely below the measurement accuracy of the FluoKin system. This had no negative effect on the precision of a final XROMM animation (see Vedio S1).

Maximal strain during walk differed from trot, which is in accordance with other studies (Table 1). It is noticeable that during trot, the tendon recoils more than during walk (its minimum is smaller during trot). This occurrence is probably due to its energy-storing function and action as a spring [2], which is defined by the main contribution of over 80% to the total energy generated by the distal forelimb [58].

Collagenase was applied to the mid-metacarpal region to induce a tendinopathy [43,50,59,60,61,62] (Figure 1). The mid-metacarpal region is prone to lesions as the crimp of the fibers begins to straighten and the fibers reach their load limit first [12,60]. Collagenase injection was also used in other biomechanical studies in horses [63,64,65,66]. Following injury, the strain behavior of the SDFT differed from its sound condition (Figure 5). The insulted mid-metacarpal region was strained less during walk and more during trot. This may be caused by weaknesses of the collagen fibers and, ultimately, the destruction of the mechanical properties due to the collagenase. This is in accordance with the findings of Dakin et al. [51], who found a decrease in stiffness in the SDFT after injury during walk. Moreover, in elastography, acute diseased tendon areas appear softer [67]. During trot, an increase in strain and in intermarker distance in the mid-metacarpal region M2 to M3 was detectable in the injured tendon (Figure 5 and Figure 6). This finding can be explained by higher velocity and impulse occurring during faster gaits in combination with insufficient viscoelastic response of the insulted tissue. Furthermore, a decrease in strain and intermarker distance in the whole metacarpal region M1 to M4 could be caused by pain-induced lameness and a resulting smaller load.

The two measurements of the right forelimb were not significantly different, and therefore, this leg was considered to be the healthy control for the left (injured) forelimb. Intermarker distances (cm) are not transferable between the right and the left forelimb due to slightly unequal implantation of the markers. Therefore, we compared the strain (%) of the two forelimbs.

In the right (sound) forelimb, the minimum and maximum intermarker distances for the whole metacarpal region M1 to M4 became larger in week 33, after the collagenase-induced insult (Figure 6). In contrast, maximum intermarker distances for the mid-metacarpal region M2 to M3 were smaller at the second measurement (two weeks post-injury) of the right forelimb. Furthermore, the strain and amplitude of tendon length variation during walk increased, whereas during trot it became smaller in week 33 in the right (sound) forelimb. Both forelimbs showed less strain during trot and a smaller walk–trot difference in the last FluoKin measurement (Figure 5), which could be an effect of the post-insult exercise protocol. As the investigated pony was untrained (small pasture and stable rest during winter) prior to study participation, it could be expected that tendon tissue responds to the mechanical stimuli of the exercise protocol with a slight adaptation process. Strain increases through exercise [68], which provides an explanation for the increase in intermarker distances seen between the two measurements in the right forelimb during walk. Despite the higher strain rates that occur when trotting, the amplitude of the intermarker distances in the second measurement, in the right forelimb, became smaller. The viscoelastic properties of tendons cause an inverse effect, due to the indirect dependence of strain and strain rate [69]. However, the effect of training should not be overestimated, since the ability to adapt is very limited in mature tendons [70]. It is not impossible, however, as a study in elderly humans proposes [71]. In the left forelimb, smaller strain rates in the final FluoKin measurement, compared to the initial measurements (three weeks after the collagenase-induced insult vs. native), could first be explained by better posture and balance of the pony, which was constantly trained during the rehabilitation phase. It seems a plausible explanation that placing the hindlimbs more underneath the body’s center of gravity releases load from the forelimb’s digital flexor tendons and thus lowers SDFT strain. Second, the stiffness and strainability of an energy-storing tendon are affected by training, which improves its viscoelastic response during movement [72,73].

The strain of the whole metacarpal region M1 to M4, compared to the mid-metacarpal region M2 to M3, shows an obvious pattern (Figure 5). The mid-metacarpal region’s contribution to the overall strain during walk is larger in sound conditions and smaller two weeks after the collagenase-induced insult. The opposite can be observed during trot: the mid-metacarpal region contributes less to the overall strain in sound conditions. This is because the tendon’s viscoelastic properties make it stiffer at high strain rates [74,75]. However, it is strained more in the left forelimb two weeks after the insult and, surprisingly, also 33 weeks after the insult (Figure 5). Therefore, we can conclude that the tendon tissue was not completely healed 33 weeks after the collagenase-induced insult and became measurable in the trotting pony. This is despite the fact that no lameness was seen and no lesion detected with ultrasound. Nevertheless, we recommend combining FluoKin with another measurement tool to be able to quantify eventually altered limb dynamics.

Stiffness increases during tendon healing [51,67], which also can be concluded from the current study, showing a decrease in strain. However, healed tendons slowly reach their initial mechanical qualities: the former defect is filled with a less loadable substitution, which has more collagen type III compared to normal tendon tissue [76]. Therefore, it continues to be predisposed to reinjury [11,46,77]. Even one year after the injury, the amount of collagen type III is increased [46,76] and the crimp pattern of the collagen fibers is altered [43,78]. Silver et al. [43] found collagen type III and shorter than normal collagen fibers up to 14 months after injury. The state can last one to three years, or until the initial condition is restored [43,79]. In chronic lesions, Crevier-Denoix et al. [80] found an “understraining” of the pathological region during tensile testing, which is explained by an accumulation of fibrous material. Additionally, they found a compensatory “overstraining” of the adjacent transitional area, which lies between the damaged and normal tissue. This is an additional explanation for the high incidence of reinjury in this region. This is in line with our findings during walk. In contrast, the strain is distributed inversely during trot (Figure 5). The injured region is strained more compared to sound and healed conditions, and the whole metacarpal region is strained less compared to the sound condition. Therefore, and because of normal clinical and ultrasonographic findings and the absence of lameness, we conclude that the lesion induced in our study was not within a chronic inflammatory stadium but in the healing process.

The use of a treadmill had no significant effect on tendon strain, although an influence on gait patterns has been reported. Stance phase is prolonged on a treadmill and limbs are moved more caudally, but placed normally [81]. Since the SDFT undergoes peak load at the early stance phase [45], treadmill usage during walk had no significant effect on strain and intermarker distances compared to walking without the treadmill.

Ponies show a different gait when trotting at the same velocity as horses [82], due in part to different distal limb and hoof conformations [83]. Therefore, the pony used in our study was not forced to an extended trot but could choose the speed of a working trot. Despite the differences, a precautious transfer of these results to horses is highly promising for better understanding of tendon biomechanics during healing. In any case, further studies will be necessary to provide more evidence for a reliable comparison of tendon biomechanics in ponies and horses.

## 5. Conclusions

In this study, tendon strain was directly examined (without needing indirect calculation) under sound, injured, and healing conditions with highly precise, biplanar, high-speed fluoroscopy (FluoKin). This technique avoids disruption of the physiological gait patterns of the animal through the examination technique and equipment itself. Our results demonstrate that implantation of tantalum beads is a valuable technique to investigate tendon strain in horses in vivo as well as tendon healing over long periods with FluoKin. The changes in intermarker distances in the sound equine SDFT reflect physiological biomechanical behavior during walk and trot. In the injured SDFT, the mid-metacarpal region contributes inversely to the strain in the metacarpal region, compared to the sound condition (i.e., a lesser contribution during walk and more during trot). Thirty-three weeks after the collagenase-induced insult, neither lameness nor lesion could be observed with an ultrasound examination. However, the FluoKin measurements during trot clearly indicated that the tendon tissue had not fully healed. For sport horses in particular, this should provide valuable information for preventing reinjury.

This study provides new insights into the biomechanical changes of the SDFT under different health conditions. We show that established techniques may not be sufficient to accurately assess the healing process of the SDFT. Nevertheless, additional cases should be studied to permit the transmission of this method to other anatomical structures or to use this technique for practical applications, such as in regenerative medicine.

## Figures and Tables

**Table 1:** Strains of the superficial digital flexor tendon (SDFT) reported in literature.

	Animal	Specimen	Strain		
			During Walk	During Trot	Rupture
Riemersma et al. 1985	ponies	whole SDFT	1.74%		
Riemersma et al. 1988	ponies	whole SDFT	2.3%		
Stephens et al. 1989	horses	whole SDFT	3.3%		
Jansen et al. 1993	ponies	whole SDFT	3.5%		
Crevier et al. 1996	horses	metacarpal segments			proximal 4.78%, mid 5.05%, distal 4.43%
Riemersma et al. 1996	ponies	whole SDFT	2.19%	4.15%	
Butcher et al. 2007	horses	whole SDFT	3.6%	5.6%	
Lawson et al. 2007	horses	whole SDFT	6.71%	8.46%	
This study	pony	metacarpal region of the SDFT	2.86% mid: 3.13%	6.78% mid: 6.06%	

## Data Availability

All data are available in the text or as Supplementary Materials.

## Supplementary Materials

The following are available online at https://www.mdpi.com/article/10.3390/vetsci8060092/s1, Video S1: Video of biplanar high-speed fluoroscopy and 3D animation.

## References

[1] Williams R.B., Harkins L.S., Hammond C.J., Wood J.L. (2001). Racehorse injuries, clinical problems and fatalities recorded on British racecourses from flat racing and National Hunt racing during 1996, 1997 and 1998. Equine Vet. J..

[2] Smith R.K.W., Birch H.L., Goodman S., Heinegård D., Goodship A.E. (2002). The influence of ageing and exercise on tendon growth and degeneration–Hypotheses for the initiation and prevention of strain-induced tendinopathies. Comp. Biochem. Physiol. Part. A Mol. Integr. Physiol..

[3] Butcher M.T., Hermanson J.W., Ducharme N.G., Mitchell L.M., Soderholm L.V., Bertram J.E.A. (2007). Superficial digital flexor tendon lesions in racehorses as a sequela to muscle fatigue: A preliminary study. Equine Vet. J..

[4] Patterson-Kane J.C., Firth E.C. (2009). The pathobiology of exercise-induced superficial digital flexor tendon injury in Thoroughbred racehorses. Vet. J..

[5] Wollenman P., McMahon P.J., Knapp S., Ross M.W., Ross M.W., Dyson S.J. (2011). Lameness in the Polo Pony. Diagnosis and Management of Lameness in the Horse.

[6] Davis C.S., Smith R.K.W., Auer J.A., Stick J.A. (2006). Diseases of tendon and ligament disorders. Equine Surgery.

[7] Dyson S.J. (2004). Medical management of superficial digital flexor tendonitis: A comparative study in 219 horses (1992–2000). Equine Vet. J..

[8] Thorpe C.T., Clegg P.D., Birch H.L. (2010). A review of tendon injury: Why is the equine superficial digital flexor tendon most at risk?. Equine Vet. J..

[9] Patterson-Kane J.C., Becker D.L., Rich T. (2012). The pathogenesis of tendon microdamage in athletes: The horse as a natural model for basic cellular research. J. Comp. Pathol..

[10] McCullagh K.G., Goodship A.E., Silver I.A. (1979). Tendon injuries and their treatment in the horse. Vet. Rec..

[11] Goodship A.E., Birch H.L., Wilson A.M. (1994). The pathobiology and repair of tendon and ligament injury. Vet. Clin. N. Am. Equine Pract.

[12] Patterson-Kane J.C., Wilson A.M., Firth E.C., Parry D.A., Goodship A.E. (1998). Exercise-related alterations in crimp morphology in the central regions of superficial digital flexor tendons from young thoroughbreds: A controlled study. Equine Vet. J..

[13] Dowling B.A., Dart A.J., Hodgson D.R., Smith R.K. (2000). Superficial digital flexor tendonitis in the horse. Equine Vet. J..

[14] Stephens P.R., Nunamaker D.M., Butterweck D.M. (1989). Application of a Hall-effect transducer for measurement of tendon strains in horses. Am. J. Vet. Res..

[15] Riemersma D.J., van den Bogert A.J., Jansen M.O., Schamhardt H.C. (1996). Tendon strain in the forelimbs as a function of gait and ground characteristics and in vitro limb loading in ponies. Equine Vet. J..

[16] Pourcelot P., Defontaine M., Ravary B., Lemâtre M., Crevier-Denoix N. (2005). A non-invasive method of tendon force measurement. J. Biomech..

[17] Duenwald S., Kobayashi H., Frisch K., Lakes R., Vanderby R. (2011). Ultrasound echo is related to stress and strain in tendon. J. Biomech..

[18] Vergari C., Ravary-Plumioën B., Evrard D., Laugier P., Mitton D., Pourcelot P., Crevier-Denoix N. (2012). Axial speed of sound is related to tendon’s nonlinear elasticity. J. Biomech..

[19] Ellison M.E., Duenwald-Kuehl S.E., Forrest L.J., Vanderby R., Brounts S.H. (2014). Reproducibility and feasibility of acoustoelastography in the superficial digital flexor tendons of clinically normal horses. Am. J. Vet. Res..

[20] Meershoek L.S., Lanovaz J.L. (2001). Sensitivity analysis and application to trotting of a noninvasive method to calculate flexor tendon forces in the equine forelimb. Am. J. Vet. Res..

[21] Crevier-Denoix N., Ruel Y., Dardillat C., Jerbi H., Sanaa M., Collobert-Laugier C., Ribot X., Denoix J.M., Pourcelot P. (2005). Correlations between mean echogenicity and material properties of normal and diseased equine superficial digital flexor tendons: An in vitro segmental approach. J. Biomech..

[22] Brainerd E.L., Baier D.B., Gatesy S.M., Hedrick T.L., Metzger K.A., Gilbert S.L., Crisco J.J. (2010). X-ray reconstruction of moving morphology (XROMM): Precision, accuracy and applications in comparative biomechanics research. J. Exp. Zool. A Ecol. Genet. Physiol..

[23] Friden T., Ryd L., Lindstrand A. (1992). Laxity and graft fixation after reconstruction of the anterior cruciate ligament. A roentgen stereophotogrammetric analysis of 11 patients. Acta Orthop. Scand..

[24] Schepull T., Kvist J., Andersson C., Aspenberg P. (2007). Mechanical properties during healing of Achilles tendon ruptures to predict final outcome: A pilot Roentgen stereophotogrammetric analysis in 10 patients. BMC Musculoskelet. Disord..

[25] Cashman P.M.M., Baring T., Reilly P., Emery R.J.H., Amis A.A. (2010). Measurement of migration of soft tissue by modified Roentgen stereophotogrammetric analysis (RSA): Validation of a new technique to monitor rotator cuff tears. J. Med. Eng. Technol..

[26] Marshall N.E., Keller R.A., Okoroha K., Guest J.M., Yu C., Muh S., Moutzouros V. (2016). Radiostereometric Evaluation of Tendon Elongation After Distal Biceps Repair. Orthop. J. Sports Med..

[27] Wagner F.C., Reese S., Gerlach K., Böttcher P., Mülling C.K.W. (2021). Cyclic tensile tests of Shetland pony superficial digital flexor tendon with an optimized cryo-clamp combined with biplanar high-speed fluoroscopy. BMC Vet. Res..

[28] Bussières G., Jacques C., Lainay O., Beauchamp G., Leblond A., Cadore J.L., Desmaizieres L.M., Cuvelliez S.G., Troncy E. (2008). Development of a composite orthopaedic pain scale in horses. Res. Vet. Sci..

[29] Smith R.K.W., McIlwraith C.W. (2012). Consensus on equine tendon disease: Building on the 2007 Havemeyer symposium. Equine Vet. J..

[30] Dalla Costa E., Minero M., Lebelt D., Stucke D., Canali E., Leach M.C. (2014). Development of the horse grimace scale (HGS) as a pain assessment tool in horses undergoing routine castration. PLoS ONE.

[31] Weiss M., Reich E., Grund S., Mülling C.K.W., Geiger S.M. (2017). Validation of 2 noninvasive, markerless reconstruction techniques in biplane high-speed fluoroscopy for 3-dimensional research of bovine distal limb kinematics. J. Dairy Sci..

[32] Geiger S.M., Reich E., Böttcher P., Grund S., Hagen J. (2018). Validation of biplane high-speed fluoroscopy combined with two different noninvasive tracking methodologies for measuring in vivo distal limb kinematics of the horse. Equine Vet. J..

[33] Knoerlein B.J., Baier D.B., Gatesy S.M., Laurence-Chasen J.D., Brainerd E.L. (2016). Validation of XMALab software for marker-based XROMM. J. Exp. Biol..

[34] De Grauw J.C., van Loon J.P.A.M. (2016). Systematic pain assessment in horses. Vet. J..

[35] Van Loon J.P.A.M., van Dierendonck M.C. (2019). Pain assessment in horses after orthopaedic surgery and with orthopaedic trauma. Vet. J..

[36] Khan K.M., Cook J.L., Bonar F., Harcourt P., Astrom M. (1999). Histopathology of common tendinopathies. Update and implications for clinical management. Sports Med..

[37] Sugg K.B., Lubardic J., Gumucio J.P., Mendias C.L. (2014). Changes in macrophage phenotype and induction of epithelial-to-mesenchymal transition genes following acute Achilles tenotomy and repair. J. Orthop. Res..

[38] Killian M.L., Cavinatto L., Galatz L.M., Thomopoulos S. (2012). The role of mechanobiology in tendon healing. J. Shoulder Elbow Surg..

[39] Alberius P. (1983). Bone reactions to tantalum markers. A scanning electron microscopic study. Acta Anat..

[40] Aronson A.S., Jonsson N., Alberius P. (1985). Tantalum markers in radiography. An assessment of tissue reactions. Skeletal Radiol..

[41] Roos P.J., Hull M.L., Howell S.M. (2004). How cyclic loading affects the migration of radio-opaque markers attached to tendon grafts using a new method: A study using roentgen stereophotogrammetric analysis (RSA). J. Biomech. Eng..

[42] Goldin B., Block W.D., Pearson J.R. (1980). Wound healing of tendon–I. Physical, mechanical and metabolic changes. J. Biomech..

[43] Silver I.A., Brown P.N., Goodship A.E., Lanyon L.E., McCullagh K.G., Perry G.C., Williams I.F. (1983). A clinical and experimental study of tendon injury, healing and treatment in the horse. Equine Vet. J. Suppl..

[44] Enwemeka C.S. (1989). Inflammation, cellularity, and fibrillogenesis in regenerating tendon: Implications for tendon rehabilitation. Phys. Ther..

[45] Denoix J.M. (1994). Functional anatomy of tendons and ligaments in the distal limbs (manus and pes). Vet. Clin. N. Am. Equine Pract..

[46] Watkins J.P., Auer J.A., Gay S., Morgan S.J. (1985). Healing of surgically created defects in the equine superficial digital flexor tendon: Collagen-type transformation and tissue morphologic reorganization. Am. J. Vet. Res..

[47] Van den Boom R., Wilmink J.M., O’Kane S., Wood J., Ferguson M.W.J. (2002). Transforming growth factor-beta levels during second- intention healing are related to the different course of wound contraction in horses and ponies. Wound Repair Regen..

[48] Wilmink J.M., van Herten J., van Weeren P.R., Barneveld A. (2002). Retrospective study of primary intention healing and sequestrum formation in horses compared to ponies under clinical circumstances. Equine Vet. J..

[49] Pickersgill C.H., Marr C.M., Reid S.W. (2001). Repeatability of diagnostic ultrasonography in the assessment of the equine superficial digital flexor tendon. Equine Vet. J..

[50] Karlin W.M., Stewart A.A., Durgam S.S., Naughton J.F., O’Dell-Anderson K.J., Stewart M.C. (2011). Evaluation of experimentally induced injury to the superficial digital flexor tendon in horses by use of low-field magnetic resonance imaging and ultrasonography. Am. J. Vet. Res..

[51] Dakin S.G., Jespers K., Warner S., O’Hara L.K., Dudhia J., Goodship A.E., Wilson A.M., Smith R.K.W. (2011). The relationship between in vivo limb and in vitro tendon mechanics after injury: A potential novel clinical tool for monitoring tendon repair. Equine Vet. J..

[52] Crevier N., Pourcelot P., Denoix J.-M., Geiger D., Bortolussi C., Ribot X., Sanaa M. (1996). Segmental variations of in vitro mechanical properties in equine superficial digital flexor tendons. Am. J. Vet. Res..

[53] Solomon L.B., Callary S.A. (2011). Emerging ideas: Soft tissue applications of radiostereometric analysis. Clin. Orthop. Relat. Res..

[54] Smith C.K., Hull M.L., Howell S.M. (2005). Migration of radio-opaque markers injected into tendon grafts: A study using roentgen stereophotogrammetric analysis (RSA). J. Biomech. Eng..

[55] Rohwedder T., Fischer M.S., Böttcher P. (2017). In vivo fluoroscopic kinematography of dynamic radio-ulnar incongruence in dogs. Open Vet. J..

[56] Rohwedder T., Fischer M.S., Böttcher P. (2018). In vivo axial humero-ulnar rotation in normal and dysplastic canine elbow joints. Tierarztl. Prax. Ausg. K Kleintiere. Heimtiere..

[57] Flanigan P., Kshettry V.R., Benzel E.C. (2014). World War II, tantalum, and the evolution of modern cranioplasty technique. Neurosurg. Focus.

[58] Harrison S.M., Whitton R.C., Kawcak C.E., Stover S.M., Pandy M.G. (2010). Relationship between muscle forces, joint loading and utilization of elastic strain energy in equine locomotion. J. Exp. Biol.

[59] Williams I.F., McCullagh K.G., Goodship A.E., Silver I.A. (1984). Studies on the pathogenesis of equine tendonitis following collagenase injury. Res. Vet. Sci..

[60] Wilmink J., Wilson A.M., Goodship A.E. (1992). Functional significance of the morphology and micromechanics of collagen fibres in relation to partial rupture of the superficial digital flexor tendon in racehorses. Res. Vet. Sci..

[61] Nabeshima Y., Grood E.S., Sakurai A., Herman J.H. (1996). Uniaxial tension inhibits tendon collagen degradation by collagenase in vitro. J. Orthop. Res..

[62] Oloumi M.M., Vosough D., Derakhshanfar A., Nematollahi M.H. (2011). The healing potential of plantago lanceolata ointment on collagenase-induced tendinitis in burros (*Equus asinus*). J. Equine Vet. Sci..

[63] Clayton H.M., Schamhardt H.C., Willemen M.A., Lanovaz J.L., Colborne G.R. (2000). Kinematics and ground reaction forces in horses with superficial digital flexor tendinitis. Am. J. Vet. Res..

[64] Clayton H.M., Schamhardt H.C., Willemen M.A., Lanovaz J.L., Colborne G.R. (2000). Net joint moments and joint powers in horses with superficial digital flexor tendinitis. Am. J. Vet. Res..

[65] Clayton H.M., Willemen M.A., Lanovaz J.L., Schamhardt H.C. (2000). Effects of a Heel Wedge in Horses with Superficial Digital Flexor Tendinitis. Vet. Comp. Orthop. Traumatol..

[66] Meershoek L.S., Lanovaz J.L., Schamhardt H.C., Clayton H.M. (2002). Calculated forelimb flexor tendon forces in horses with experimentally induced superficial digital flexor tendinitis and the effects of application of heel wedges. Am. J. Vet. Res..

[67] Lustgarten M., Redding W.R., Labens R., Davis W., Daniel T.M., Griffith E., Seiler G.S. (2015). Elastographic evaluation of naturally occuring tendon and ligament injuries of the equine distal limb. Vet. Radiol. Ultrasound.

[68] Bukowiecki C.F., Bramlage L.R., Gabel A.A. (1987). In vitro strength of the suspensory apparatus in training and resting horses. Vet. Surg..

[69] Wang J.H.C. (2006). Mechanobiology of tendon. J. Biomech..

[70] Smith R.K.W., Goodship A.E. (2008). The effect of early training and the adaptation and conditioning of skeletal tissues. Vet. Clin. N. Am. Equine Pract.

[71] Narici M.V., Maganaris C.N. (2006). Adaptability of elderly human muscles and tendons to increased loading. J. Anat..

[72] Fouré A., Nordez A., Cornu C. (2010). Plyometric training effects on Achilles tendon stiffness and dissipative properties. J. Appl. Physiol..

[73] Kubo K., Ikebukuro T., Maki A., Yata H., Tsunoda N. (2012). Time course of changes in the human Achilles tendon properties and metabolism during training and detraining in vivo. Eur. J. Appl. Physiol..

[74] Lochner F.K., Milne D.W., Mills E.J., Groom J.J. (1980). In vivo and in vitro measurement of tendon strain in the horse. Am. J. Vet. Res..

[75] Johnson G.A., Livesay G.A., Woo S.L., Rajagopal K.R. (1996). A single integral finite strain viscoelastic model of ligaments and tendons. J. Biomech. Eng..

[76] Williams I.F., Heaton A., McCullagh K.G. (1980). Cell morphology and collagen types in equine tendon scar. Res. Vet. Sci.

[77] Parry D.A., Barnes G.R., Craig A.S. (1978). A comparison of the size distribution of collagen fibrils in connective tissues as a function of age and a possible relation between fibril size distribution and mechanical properties. Proc. R Soc. Lond. B Biol. Sci..

[78] Diamant J., Keller A., Baer E., Litt M., Arridge R.G. (1972). Collagen; ultrastructure and its relation to mechanical properties as a function of ageing. Proc. R Soc. Lond. B Biol. Sci..

[79] Stashak T.S., Theoret C. (2008). Equine Wound Management.

[80] Crevier-Denoix N., Collobert C., Pourcelot P., Denoix J.M., Sanaa M., Geiger D., Bernard N., Ribot X., Bortolussi C., Bousseau B. (1997). Mechanical properties of pathological equine superficial digital flexor tendons. Equine Vet. J. Suppl..

[81] Buchner H.H., Savelberg H.H., Schamhardt H.C., Merkens H.W., Barneveld A. (1994). Kinematics of treadmill versus overground locomotion in horses. Vet. Q..

[82] Back W., Schamhardt H.C., van Weeren P.R., Barneveld A. (1999). A comparison between the trot of pony and horse foals to characterize equine locomotion at young age. Equine Vet. J. Suppl..

[83] Crevier-Denoix N., Ravary-Plumiöen B., Vergari C., Camus M., Holden-Douilly L., Falala S., Jerbi H., Desquilbet L., Chateau H., Denoix J.M. (2013). Comparison of superficial digital flexor tendon loading on asphalt and sand in horses at the walk and trot. Vet. J..

